# AST: An Automated Sequence-Sampling Method for Improving the Taxonomic Diversity of Gene Phylogenetic Trees

**DOI:** 10.1371/journal.pone.0098844

**Published:** 2014-06-03

**Authors:** Chan Zhou, Fenglou Mao, Yanbin Yin, Jinling Huang, Johann Peter Gogarten, Ying Xu

**Affiliations:** 1 Computational Systems Biology Laboratory, Department of Biochemistry and Molecular Biology and Institute of Bioinformatics, University of Georgia, Athens, Georgia, United States of America; 2 Department of Biology, East Carolina University, Greenville, North Carolina, United States of America; 3 Department of Molecular and Cell Biology, University of Connecticut, Storrs, Connecticut, United States of America; 4 College of Computer Science and Technology, Jilin University, Changchun, China; Belgian Nuclear Research Centre SCK•CEN, Belgium

## Abstract

A challenge in phylogenetic inference of gene trees is how to properly sample a large pool of homologous sequences to derive a good representative subset of sequences. Such a need arises in various applications, e.g. when (1) accuracy-oriented phylogenetic reconstruction methods may not be able to deal with a large pool of sequences due to their high demand in computing resources; (2) applications analyzing a collection of gene trees may prefer to use trees with fewer operational taxonomic units (OTUs), for instance for the detection of horizontal gene transfer events by identifying phylogenetic conflicts; and (3) the pool of available sequences is biased towards extensively studied species. In the past, the creation of subsamples often relied on manual selection. Here we present an Automated sequence-Sampling method for improving the Taxonomic diversity of gene phylogenetic trees, AST, to obtain representative sequences that maximize the taxonomic diversity of the sampled sequences. To demonstrate the effectiveness of AST, we have tested it to solve four problems, namely, inference of the evolutionary histories of the small ribosomal subunit protein S5 of *E. coli*, 16 S ribosomal RNAs and glycosyl-transferase gene family 8, and a study of ancient horizontal gene transfers from bacteria to plants. Our results show that the resolution of our computational results is almost as good as that of manual inference by domain experts, hence making the tool generally useful to phylogenetic studies by non-phylogeny specialists. The program is available at http://csbl.bmb.uga.edu/~zhouchan/AST.php.

## Introduction

Reconstruction of gene trees represents a commonly encountered problem in evolutionary studies, such as inferring the evolutionary history of a gene (or a gene family) [1], [2], finding the origin of a gene, discovering the function of a gene [3], [4], and estimating species trees from gene trees [5], [6], [7], [8], [9]. Reconstructing the phylogenetic history of a gene (or gene family) generally involves three steps: 1) selection of homologous sequences (DNA, RNA, or protein sequences); 2) multiple sequence alignment (MSA); and 3) phylogenetic tree reconstruction. Selection of homologous sequences is one of the key steps [10], [11], [12], [13]. Different strategies of sequence sampling may lead to different gene trees. The problem of sequence sampling for gene tree construction has been generally treated in a subjective manner, although the related problem of taxonomic sampling for determining species phylogenies has been extensively discussed and investigated in the past two decades [14], [15], [16], [17], [18], [19], [20], [21]. Taxonomic sampling of species trees refers to sampling of taxa based on some genetic markers of taxa or whole genomes, rather than sequences of genes or proteins [22], [23]. In this work, we study the sequence sampling problem for gene trees.

One reason for the necessity of sequence sampling is the rapidly increasing amount of genomic data due to the advancement of next-generation sequencing techniques. A huge dataset of homologous genes (e.g. 537,686 sequences of glycosyl transferase gene family 2 [24]) may prevent biologists from using accuracy-oriented MSA software tools, such as Muscle [25], Mafft [26], ClusterW [27], T-Coffee [28], SAT-é [29], [30], and PRANK [31], [32], [33], and phylogenetic tree estimation methods, such as PhyML [34], MrBayes [35] and PhyloBayes [36], due to their high demands for computational resources, including both memory and time. Even software tools specifically designed for large datasets have to limit the size of an input dataset. For example, DACTAL [37] is shown to be useful for datasets up to 28, 000 sequences, but the accuracy of alignments and associated trees decreases as the number of sequences increases.

An additional reason for sequence sampling is to facilitate the detection of horizontal gene transfer (HGT) based on phylogenetic tree comparisons. This is because many phylogenetic tree-based HGT detection approaches will be more applicable and accurate when smaller gene trees are used [38], [39], [40].

The problem of homologous sequence sampling for a subset of sequences with diverse coverage is not trivial owing to the current biased pool of sequenced genomes [41] i.e. model organisms and medically (or economically) important species tend to have more sequences in the current databanks than others. Simple-minded sampling strategies may lead to sequence datasets that are biased towards certain families of organisms.

Two general strategies have been adopted in sequence sampling when calculating gene phylogenies. One is to sample the most similar sequences (SS) of a query sequence, using either BLAST [42] or HMM-based methods [43]; another is manual selection (MS), which samples sequences based on phylogenetic expertise and knowledge about the evolutionary relationships among relevant organisms. That knowledge may come from a preliminary tree with as many homologous sequences as possible, or from domain experts, who manually sample the tree-associated sequences based on their expertise and experiences. The use of SS may generate a tree that lacks important lineages as a result of the low coverage of some taxonomic branches, while MS, although perhaps suitable for limited case studies, is not generally scalable. Here we focus on the question of sequence sampling to achieve a high taxonomic diversity.

To obtain gene trees with high taxonomic diversity, we developed an algorithm named as ***AST*** to **a**utomatically **s**elect representative homologous sequences over **t**axa. In this study we show that, for the same number of sampled sequences, AST gives rise to more diverse taxa as compared to the currently used methods.

To illustrate its effectiveness, we applied the AST method to resolve the following evolutionary questions: (i) can we infer the evolutionary history of the small ribosomal subunit (SSU) protein S5 (rpS5), 16 S ribosomal RNA (16 S rRNA) and glycosyl-transferase gene family 8 (GT8), and can we identify ancient HGTs from bacteria to eukaryotes.

## Materials and Methods

### 2.1 Sampling algorithm

**Figure 1:**
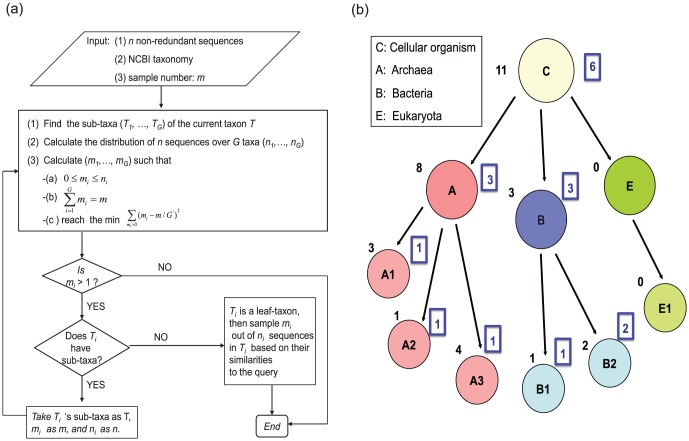
AST algorithm. (a) Workflow of the AST algorithm. (b) An example of the sampling procedure of AST. Each circle represents one taxon: C-all *Cellular Organism*; A-*Archaea*; B-*Bacteria*; A1 is an archaeal taxon labeled as A1, similar for A2, A3, B1, and B2. The number listed on the left shoulder of the circle (outside the rectangle) is the number of sequences from the taxon labeled in the circle, and the number listed on the right shoulder of each circle is the number of sampled sequences by AST from the taxon in the circle. In this example there are a total of 11 homologous sequences in all cellular organisms, among which 8 belong to archaea, 3 from bacteria and none from eukaryotes.

The AST algorithm samples *m* sequences from *n* non-redundant homologous sequences of a query sequence, based on the NCBI taxonomic distribution covered by the *n* sequences. AST ensures that the sampled sequences will have a high taxonomic diversity covered by the given pool of *n* homologous sequences and the most even distribution across taxa, *m<n*. Consider a taxon *T* having *G* sub-taxa {*T_1_, T_2_…, T_G_*}with *T_i_* having *n_i_* homologous sequences (to the query) so 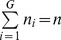, *n_i_≥0*. Here, the sub- taxa {*T_1_, T_2_…, T_G_*}are the children taxa of a taxon *T*, rather than all its descendants. The goal is to select *m_i_* representatives from the *n_i_* homologs from *T_i_* such that *m_i_* will be chosen to be the integer value that is closest to the average number of sequences (calculated as *m/G′*) in each taxon among the *G′* taxa, where *G′* is the number of taxa having non-zero *n* homologs (*n*>0) of the query. Then all the {*m_1_, m_2_, m_3_*,… *m_G′_*} values will be the same value or differ by 1 and all the remaining *m_i_*: { *m_G′+1_*,…, *m_G_*} where taxa *T_i_* does not have homologs (i.e. *n_i_* = 0) are set to be zero (see Figure 1b as an example). Explicitly, if *m/G′* does not equal an integer, we sort the taxa descendingly by number of sequences in each taxon, then assign *m_i_* as int*(m/G′)+1* for the first *m-G′× int(m/G′)* taxa, as int(*m/G′*) for the remaining taxa, where int(*x*) represents the integral part of *x*. It is easy to check that 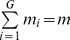.

To maximize the taxonomic diversity, our algorithm searches exclusively among the hierarchy of NCBI taxa. It recursively solves this problem as follows. If *m_i_*>1 and *T_i_* has children taxa in the next level, then recursively call the above algorithm by setting *n* to *n_i_, m* to *m_i_* and *T_i_* to the current taxon. If *m_i_*>1 and *T_i_* does not have sub-taxa (i.e., a leaf-taxon), sample *m_i_* sequences with the highest sequence similarity to the query from the *n_i_* sequences. The algorithm iterates until *m_i_* = 1 or 0, or *T_i_* becomes a leaf-taxon. Figure 1a illustrates a workflow of the algorithm (we refer readers to Figure 1b for an example).

### 2.2 Simulated data

We used the EvolveAGene [44] program to generate three types of simulated gene trees: symmetric trees, random trees and asymmetric trees, as each of these three types may exist in reality. EvolveAGene [44] can simulate the evolution of DNA sequences through mimicking mutation and natural selection, and generate the off-spring sequences, whose ultimate structures will be symmetric or random trees based on specified parameters. Here we take each simulated DNA sequence in each generation as the whole genome of an organism, with each node of the simulated trees as a taxon. The relationships among the taxa (nodes) are available from the output of EvolveAGene.

To generate the simulated trees for this study, we randomly chose the *xisC* gene of bacterium *Nostoc sp. PCC 7120* (GenBank accession: U08014) as the initial root sequence and generated 1,024 simulated sequences using the program, where 1,024 is used because it is a power of 2 as required for generating a symmetric tree, and this size is comparable to the order of magnitude of the number of currently sequenced genomes [45].

EvolveAGene provides an option for generating random trees without any specified tree topology. When using this program, we set the average branch length at 0.3 and the number of leaf taxa as 1,024 with all the other parameters set at their default values.

To generate asymmetric trees, we first generated a symmetric tree with 2,048 leafs, and then select 1,024 leafs to construct an asymmetric tree using the following procedure. We randomly chose *x* percentage of the selected 1,024 leafs from the left branch of the symmetric tree and (1.0−*x*) percentage of 1,024 leafs from the right branch. Here we used *x* values equal to 0.1, 0.2, 0.3, and 0.4 to generate the trees.

### 2.3 Biological data

Amino acid sequences of the rpS5 proteins from 816 bacteria and 68 archaea were downloaded from the NCBI curated Protein Cluster DB (Oct, 2010) (http://www.ncbi.nlm.nih.gov/proteinclusters). They were identified as non-redundant homologs of the *Escherichia coli* rpS5 protein using pBLAST with an *E*-value <0.01. The rank of the similarity scores between these homologous proteins were based on the BLAST bitscore.

The 918 GT8 protein sequences and their pair-wise similarity scores were provided by the authors of [1].

### 2.4 Phylogenetic analyses

To construct the phylogeny for rpS5 of *E. coli* and other related organisms, we performed multiple sequence alignments using MAFFT (version 6.603) [46], employing the L-INS-I model, which adopts local pair-wise alignments by the Smith-Waterman algorithm and is considered to be one of the most accurate multiple sequence alignment methods currently available [47], [48]. Then a phylogenetic tree was constructed using the FastTree program (version 2.1.3) [49], which implements a superfast but fairly accurate approximate maximum likelihood method [49].

To study the phylogeny and horizontal gene transfer in the class-I of glycosyl-transferase gene family 8 (GT8), we adopted a rigorous PhyML [50] analysis as used in previous analyses of GT8 [1]. For the PhyML analyses, trees were built with the JTT substitution model [51] along with the following parameters: estimated proportion of invariable sites, four rate categories, estimated gamma distribution, and optimized starting BIONJ tree [52]. Bootstrapping was performed using 100 replications. MrBayes [53] analyses were used with a mixed amino acid model estimated in the run, an estimated proportion of invariable sites, an estimated gamma distribution parameter, and one million of generations.

### 2.5 AST software package

Currently, two versions of the AST program are provided at http://csbl.bmb.uga.edu/~zhouchan/AST.php a basic version, and a more advanced version. The basic AST suite consists of the core method of AST and deals with a user pre-prepared input file with the following information: a list of IDs of non-redundant homologous sequences, their taxon IDs and similarity scores. The advanced suite does not require a prepared list of IDs of homologs. Instead, it only needs the BLAST report (in xml format, -m7 output) and will generate the input file based on the BLAST report automatically. The program has a set of default parameters, such as the BLAST bitscore and *E*-value cutoffs, but users can adjust these values if needed (see the README file of the program package for details).

## Results

We assessed the performance of AST on both simulated and real biological data, and compared the results of AST with those by SS, random sampling (RS) methods and MS (that is, if results were available in the literatures [1], [24] using the MS method). Here, the SS method samples *m non-redundant* homologous sequences that are most similar to the query, while the RS method randomly samples *m non-redundant* homologous sequences. In this study we show that the trees generated by AST indeed have more taxonomic diversities than those by SS and RS, and are comparable with the taxonomic diversity of the whole gene trees that are generated with all available homologs.

### 3.1 Comparative analyses of tree construction on simulated data

We compared the taxonomic coverage of sequences sampled by AST, SS, and RS on three types of simulated trees: symmetric, random, and asymmetric trees. One hundred trees were generated for random trees and for each of the asymmetric trees with a bias index *x* = 0.1, 0.2, 0.3, 0.4, respectively (see Materials and Methods for details). Only one symmetric tree is generated since such trees always have the same topology.

The following summarizes the performance of the three methods on asymmetric trees in terms of the taxonomic coverage at each taxonomic level when sampling with *m* = 50, 100, 200, 300, 400, 500 from *n* = 1,024 sequences which are the simulated non-redundant homologs of the root-sequence (see Materials and Methods for details). Here *taxonomic level* refers to the level (the relative position) in a taxonomic hierarchy with the root taxon being at level 1, the direct children taxa being at level 2 and so on.

Here we use the asymmetric trees with bias index *x* = 0.1 as an example. Table 1 summarizes the taxonomic coverage for sub-trees as well as the whole tree at the 8^th^ taxonomic level by the three methods. The sub-trees sampled by AST cover significantly more taxa than those sampled by RS (*P*-value  = 0.025) and SS (*P*-value = 0.0017), respectively, across all the *m* values defined above (Table 1), as determined using Mann-Whitney tests. In addition, when the number of sampled sequences is larger than 200, the sub-trees sampled by AST cover all the taxa (∼116) at the 8^th^ taxonomic level of the whole tree whereas the RS and SS miss large numbers of taxonomic lineages (Table 1). Similar comparative results were obtained at all the other taxonomic levels, except for the 1^st^ and 2^nd^ levels where all the three methods sampled all the taxa. Highly similar comparative performances were observed on asymmetric trees generated using bias index *x* = 0.2, 0.3 and 0.4.

**Table 1:** The coverage over taxa at taxonomic level 8 for asymmetric trees with sequences sampled by AST, RS and SS using bias index  = 0.1.

*m*	AST a	RS b	SS c	*All-seq* d
50	49.98±0.14	37.84±2.26	4±0	*116.4±2.29*
100	96.55±1.51	59.23±3.42	7.32±0.47	
200	116.4±2.29	78.7±3.20	14.30±0.46	
300	116.4±2.29	88.22±3.30	21.26±0.46	
400	116.4±2.29	94.24±3.45	28.23±0.44	
500	116.4±2.29	99.55±3.13	35.14±0.40	

a AST: ***a***utomated ***s***ampling homologs over ***t***axa.

b SS: ***s***ampling homologs most ***s***imilar to the query by a custom script.

c RS: ***r***andom ***s***ampling by a custom script.

d All-seq: all the 1024 homologous sequences without sampling.

The first column *m* represents the number of sampled sequences and the remaining value represents the mean±std of the number of covered taxa by each method when sampling *m* sequences. The last column indicates the average along with standard deviations of the number of taxa covered by the number of all homolgous sequences for 100 asymmetric trees.

On the symmetric and random trees, we also obtained highly comparative performance results (see Table S1 and S2): in all cases the sub-trees sampled by AST cover more taxa than those sampled by SS and RS.

### 3.2 Inference of the evolutionary history of a gene or gene family

Inference of the evolutionary history for a gene (or gene family) can help to derive its detailed functions (e.g. orthologs *vs* paralogs), as well as its possible origin. In the following, we show that the global phylogenies of the *E. coli* rpS5 and 16 S bacterial ribosomal RNAs inferred based on the sub-tree sampled with AST are very similar to that inferred by the tree built on all homologs, highlighting the ability of our method to preserve the key evolutionary information of the whole phylogeny in a smaller tree. We also did a similar analysis on cell wall synthesis-related glycosyl-transferase family 8 (GT8), and the same level of high-quality phylogeny was obtained.

#### 3.2.1 Comparative analyses of the rpS5 trees by three methods

A total of 884 rpS5 proteins (i.e. 816 and 68 of bacterial and archaeal origin, respectively) were identified as non-redundant homologs of the *E. coli* rpS5 protein (see Materials and Methods). To demonstrate that AST generates a pool of more taxonomically diverse representatives than the other two methods, we compared the trees with sequences sampled by AST, SS and RS, with the whole tree based on all 884 sequences.

**Figure 2:**
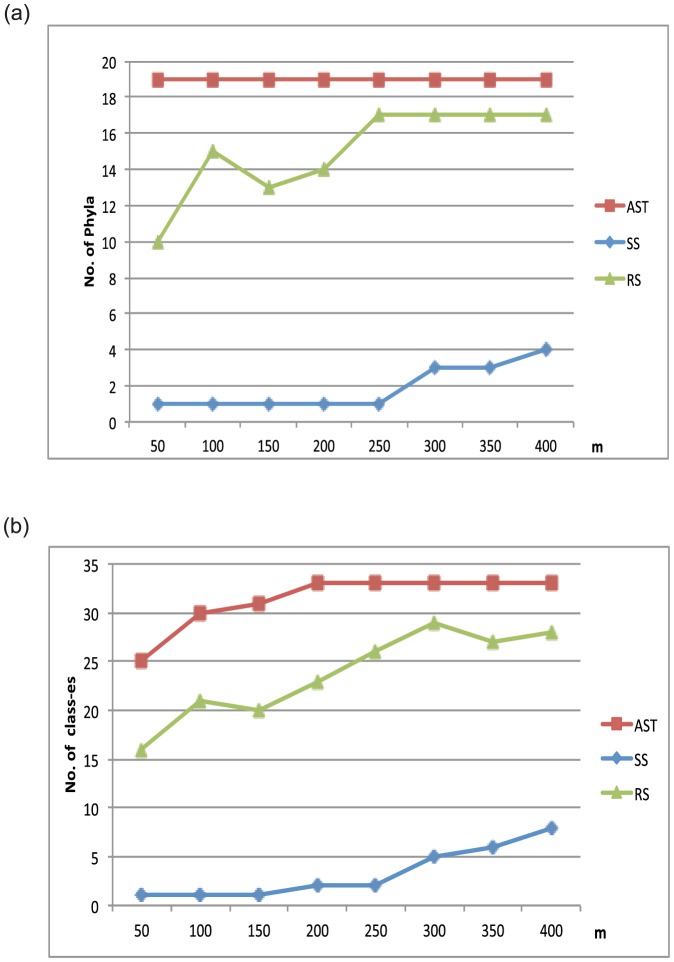
Taxonomic distributions at the phylum (a) and class level (b) for sub-trees of the rpS5 sequences sampled by AST, SS, and RS, respectively. The y-axis gives the number of phyla/classes covered by the sampled sequences, and the x-axis represents the number of sampled sequences *m*. The original non-redundant set covers 19 phyla and 33 classes (see Section 3.2.1 for details).

We note that the sub-trees sampled by AST reflect the whole tree much better than the sub-trees sampled by RS and SS. Specifically, the sub-trees built on sequences sampled using AST contain all the 19 phyla represented in the whole tree (Figure 2 and Table S3) when *m* ranges from 50 to 400. In contrast, the sub-trees sampled by RS contain 10 out of 19 phyla and the sub-tree sampled by SS only covers one of 19 phyla at *m* = 50.

It is worth noting that the advantage of AST becomes more obvious when measuring the coverage at the higher taxonomic levels (e.g. phylum (Figure 2a), class (Figure 2b) and order levels). Regardless of the number of sequences sampled, AST always gives rise to higher taxa coverage than the other two methods (Table S3).

Additionally, we evaluated the performances of AST on a published large empirical benchmark datasets for phylogeny estimation [54], which includes 38,905 16 S rRNA sequences of 34,917 bacteria. The results of the 16 S rRNA sampling (see Figure S1 and Table S4) are similar to that of the rpS5 protein.

#### 3.2.2 Inferring the evolution of the GT8 gene family

The GT8 family is a large gene family with extensive gene duplications [55]. It has been shown to fall into three well-delineated functional classes, which have cyanobacterial sequences mixed with eukaryotic sequences [1]. Here we applied AST to do the phylogenetic analysis of the same set consisting of 918 GT8 protein sequences as in [1] to examine if the same result can be achieved using this simple procedure.

**Figure 3:**
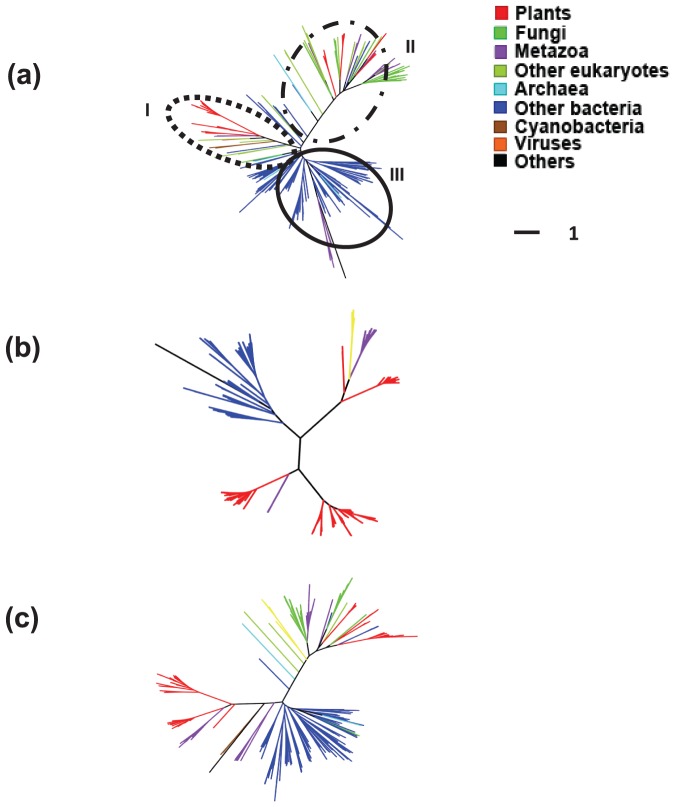
Phylogeny of 300 GT8 sequences sampled by (a) AST, (b) Similarity Sampling (SS) and (c) Random Sampling (RS) approaches, respectively. Three major functional classes are identified in (a), which is consistent with a previous publication [1]. In (b), the phylogenetic tree is composed of four classes, which are incorrect. In (c), cyanobacterial sequences (in sienna, GenBank gi: 254421706, 254423034, and 81299339) are incorrectly grouped with the out-group (in black). Color definitions: red for plants, green for fungi, purple for metazoa, olive-green for other eukaryotes, cyan for archaea, blue for other bacteria, sienna for cyanobacteria, orange for viruses and black for others.

Figure 3 shows three phylogenetic trees based on AST, SS and RS sampling for *m* = 300 out of the 918 sequences. We can see that the AST tree (Figure 3a) is very similar to the Figure 2 published in [1]. Specifically, the AST tree classifies GT8 proteins into three well-delineated functional classes and class-I has the cyanobacterial sequences mixed with eukaryote ones, while the SS and RS trees exhibit large discrepancies with the trees in [1] (not shown here). Specifically, the SS tree (Figure 3b) has four rather than three classes. It consists of only homologs in plant (in red) and bacteria (excluding cyanobacteria in sienna), but misses considerable amount of information from other taxonomic lineages such as fungi (in green), virus (in yellow), metazoa (in purple) and cyanobacteria (in sienna). The RS tree covers more lineages (Figure 3c) than the SS tree, but it does not always include cyanobacteria in class-I. This is because each random sampling procedure gave rise to different RS trees and some RS trees may group cyanobateria in the class I while others may not include any cyanobateria.

We note that the tree structure with sequences sampled by AST is highly stable for *m*> = 50. The AST trees with *m* = 50, 100, 200 and 400 are given in Figures S2–S5.

### 3.3 Detection and rigorous testing of ancient HGT events

Ancient HGTs cannot be easily detected using a phylogenetic method due to lack of high-quality datasets. Here we applied the AST method to the detection of an ancient HGT from the ancestor of cyanobacteria to the ancestor of plants [1] to show that the AST method can reliably detect HGT events and provide a rigorous test through comparing the obtained results to those that were manually derived by domain experts.

When inferring the evolutionary history of GT8, our AST tree (Figure 3a) already shows that three cyanobacterial GT8 sequences appear among eukaryotic GT8 sequences. The cyanobacterial sequences in class-I are basal to sequences from plants and some other eukaryotic GT8 proteins. This observation suggests that either there is an ancient HGT from cyanobacteria to eukaryote or cyanobacteria acquired their homolog from a eukaryote. To test these two hypotheses, a previous publication [1] manually selected 15 representative sequences based on the authors' prior knowledge, then constructed the phylogenetic trees of these 15 sequences through a computational procedure consisting of bootstrap and Bayesian analyses using PhyML [50] and MrBayes [53]. They found that in these well resolved phylogenetic trees the cyanobacterial sequence is indeed mixed with eukaryotes in class-I and groups the base of the plant homologs [1].

**Figure 4:**
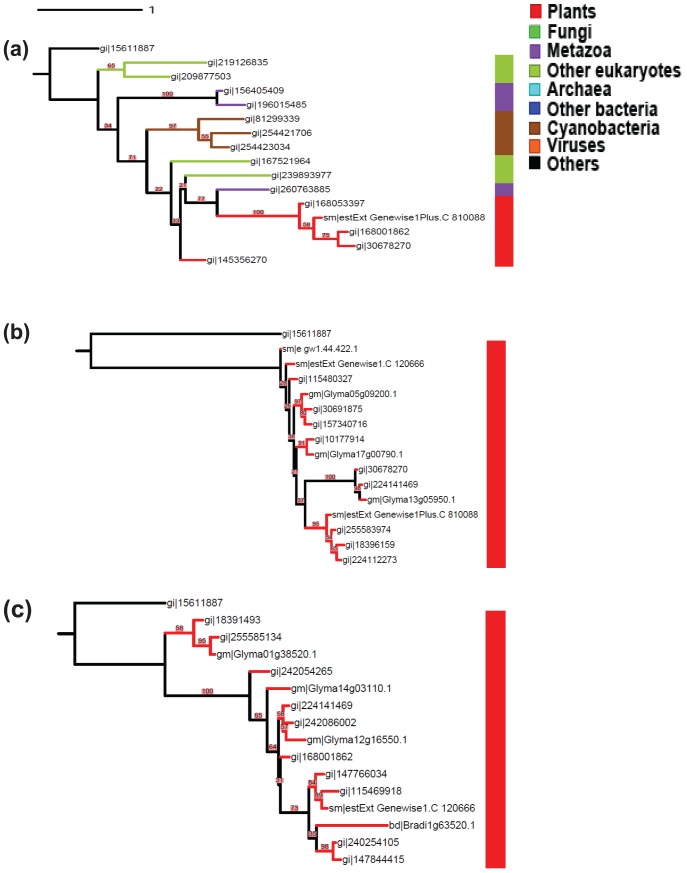
Phylogeny of 15 representative amino acid sequences from the GT8 class-I. To determine the roots of trees, we randomly selected a non-GT8 sequence (GI: 15611887) as an outgroup (in black). The sequences were sampled by (a) AST; (b) Random Sampling; and (c) Similarity Sampling approaches. The Bayesian posterior probability in grouping the 3 cyanobacterial sequences with metazoa and plants in (a) is 0.99 by using MrBayes. Tree (a) reflects the diversity of class-I with 15 sequences and also indicates a potential transfer between cyanobacteria and eukaryotes, while trees in (b) and (c) are only composed of plant sequences.

We directly applied AST to sample 15 sequences from all 268 sequences in class-I without any prior knowledge and complex computational procedure, and then used PhyML and MrBayes to perform bootstrap and Bayesian analyses with the same criteria as in [1]. The two phylogenetic trees, each generated by PhyML or MrBayes, are quite similar, although branch lengths and the statistical supports of some nodes are different (Figure 4a). The AST-based tree clusters all three cyanobacterial GT8 proteins (in sienna) and clusters them with the other class-I proteins from plants (in red) and other eukaryotes (in mignonette), except for some metazoan proteins (in purple), with strong statistical support values. This result is consistent with the results given in [1]. With regard to the RS- and SS-based trees (Figure 4b and 4c), no information about a possible HGT from cyanobacteria can be derived from either of them. AST is able to help rigorous HGT detection since the small trees sampled by AST will cover more taxa branches and hence will also cover the divergent recipients of HGT events, e.g. cyanobacteria in Figures 3 and 4.

## Discussions

In addition to the aforementioned examples, AST can also be applied to infer local phylogenies of gene trees and detect recent HGTs. For the former analyses AST requires a well-prepared file as input: a list of IDs of homologs, which should be limited within the local taxonomic lineage under consideration. For example, if one would like to study the evolution of a gene across eukaryotes, it would be reasonable to prepare a list of IDs of homologous sequences mainly from eukaryotic lineages, then AST will sample most sequences from eukaryotic and a few sequences from bacteria and/or archaea as out-groups if few (e.g. 1 or 2) of bacterial or archaeal sequences are included in the list of homologs. The detection of recent HGTs does not require sampling of homologs over all taxa. In a similar fashion as for the local gene phylogenies, AST requires a list of IDs of homologs within the concerned taxonomic group. For example, to infer recent HGTs within the proteobacteria group, only the homologs from the proteobacteria group are included in the input file. To detect ancient HGTs between distant organisms, which indeed requires sampling sequences over extensive taxa, the input file (a list of IDs of the homologs) should include homologs from all domains; otherwise the gene trees could not include branches from distant organisms which may have conflicts with their corresponding species tree, hence indicating a putative HGT.

If studying a gene family with many domain rearrangements, we suggest using the domain as a query to determine the homologous sequences instead of the entire gene sequence, and then apply the AST software to the homologous pool defined by that domain.

AST is designed to maximize taxonomic diversity, treating each branch equally and considering the topology of gene trees. In contrast, phylogenic diversity evaluates the quality of both branch length and topology [56]. If branch length is incorporated into the sampling procedure, then maximizing taxonomic diversity will also maximizing phylogenetic diversity [56], [57] of gene trees. To expand applications of the AST software, we plan to introduce weights and branch lengths (as parameters) into the sampling procedure in a future upgrade, and will also take the uneven distribution of available sequences across different taxa into consideration. Last but not the least, while AST ensures a high taxonomic diversity, it still requires strong methods for multiple sequence alignment and tree estimation for reliable gene phylogenetic inference.

## Supporting Information

Figure S1 Taxonomic distributions at the phylum (a) and class level (b) for sub-trees of 16 s ribosomal RNA sequences sampled by AST, SS, and RS, respectively. The *y*-axis gives the number of phyla/classes covered by the sampled sequences, and the *x*-axis represents the number of sampled sequences *m*. There are 26 phyla and 37 classes covered by the original non-redundant set and AST sampled sequences from each of all 26 phyla and each of all 37 classes.
(PDF)

Figure S2 Phylogenetic trees of 50 GT8 sequences sampled by AST, SS, and RS respectively. See the legend of Figure 3 for further details.
(PDF)

Figure S3 Phylogenetic trees of 100 GT8 sequences sampled by AST, SS, and RS respectively. See the legend of Figure 3 for further details.
(PDF)

Figure S4 Phylogenetic trees of 200 GT8 sequences sampled by AST, SS and RS respectively. See the legend of Figure 3 for further details.
(PDF)

Figure S5 Phylogenetic trees of 400 GT8 sequences sampled by AST, SS and RS respectively. See the legend of Figure 3 for further details.
(PDF)

Table S1 Taxonomic distributions for symmetric trees when sequences were sampled by AST, SS and RS, respectively.
(XLSX)

Table S2 Taxonomic distributions for random trees when sequences were sampled by AST, SS and RS, respectively.
(XLSX)

Table S3 Taxonomic distributions for rpS5 proteins with sequences sampled by AST, SS and RS at the super-phylum, order, family, genus, and species levels.
(XLSX)

Table S4 Taxonomic distributions for 16 S rRNA sequences with sequences sampled by AST, SS and RS at the super-phylum, order, family, genus, and species levels.
(XLSX)
