# Supplementary figures and images for: AST: An Automated Sequence-Sampling Method for Improving the Taxonomic Diversity of Gene Phylogenetic Trees
Source: PLoS One. 2014 Jun 3;9(6):e98844. doi: 10.1371/journal.pone.0098844 (PMC4044049; doi:10.1371/journal.pone.0098844)

(a)

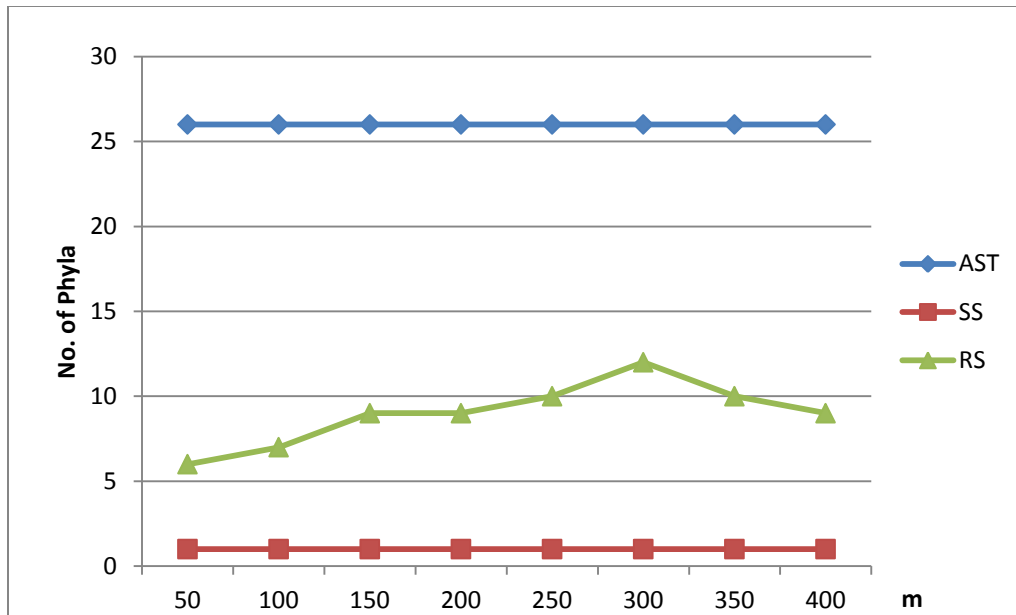

(b)

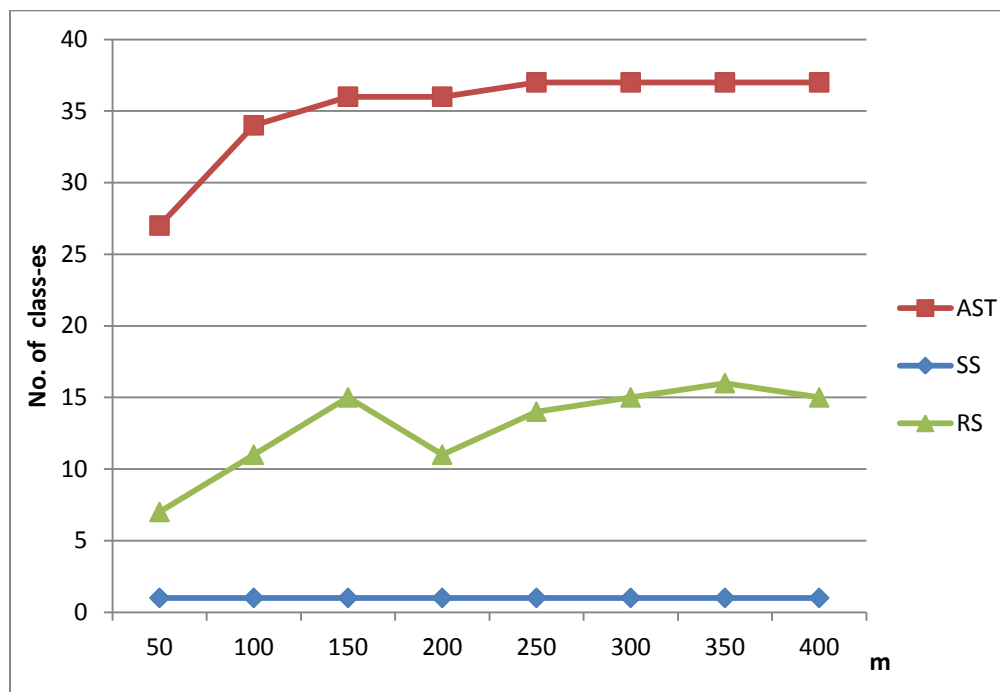

Supplement: Figure S1 — Taxonomic distributions at the phylum (a) and class level (b) for sub-trees of 16 s ribosomal RNA sequences sampled by AST, SS, and RS, respectively. The y-axis gives the number of phyla/classes covered by the sampled sequences, and the x-axis represents the number of sampled sequences m. There are 26 phyla and 37 classes covered by the original non-redundant set and AST sampled sequences from each of all 26 phyla and each of all 37 classes. (PDF) [file pone.0098844.s001.pdf]

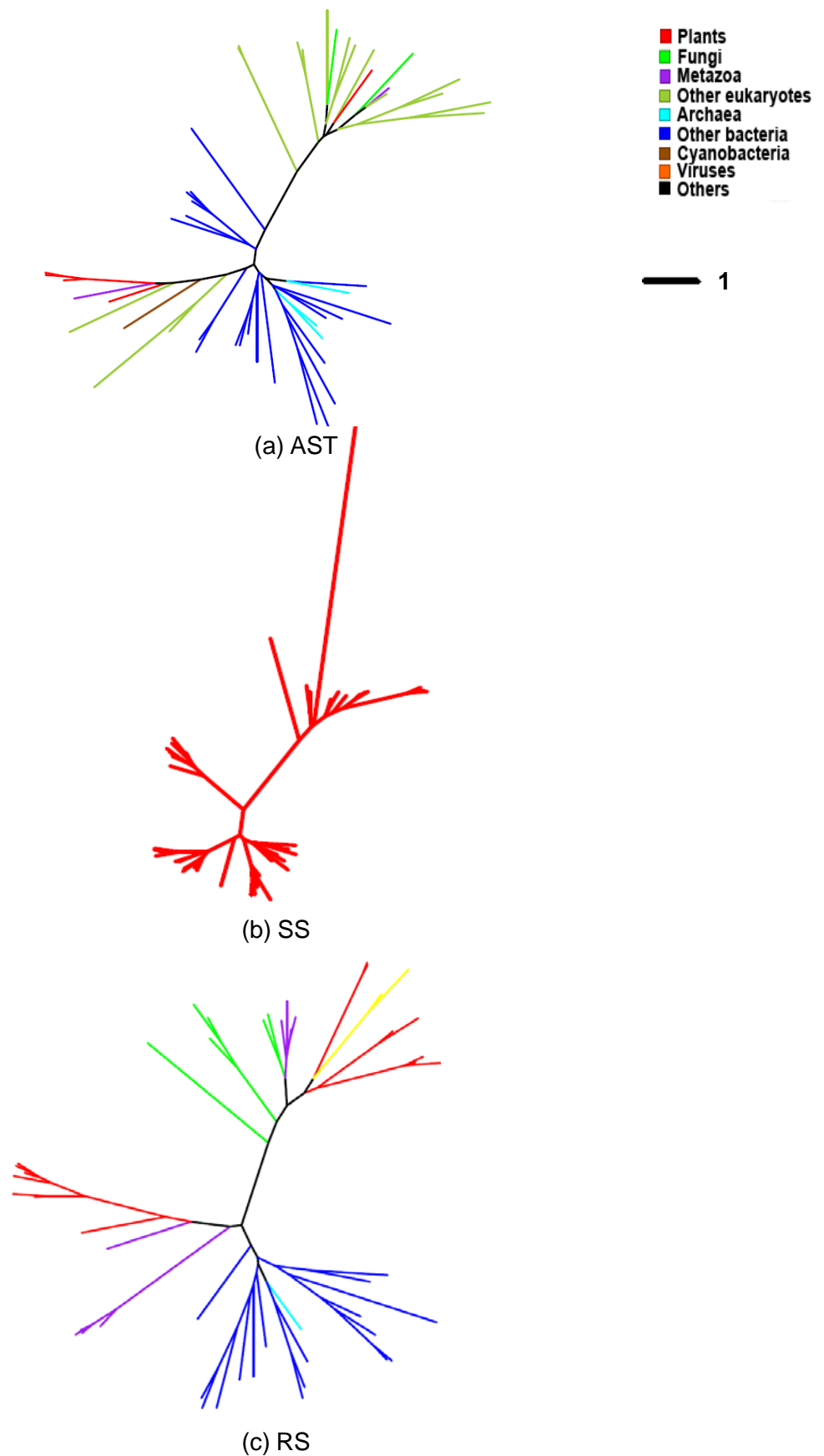

Figure S1: Phylogenetic trees of 50 GT8 sequences sampled by (a) AST, (b) SS and (c) RS, respectively.

Supplement: Figure S2 — Phylogenetic trees of 50 GT8 sequences sampled by AST, SS, and RS respectively. See the legend of Figure 3 for further details. (PDF) [file pone.0098844.s002.pdf]

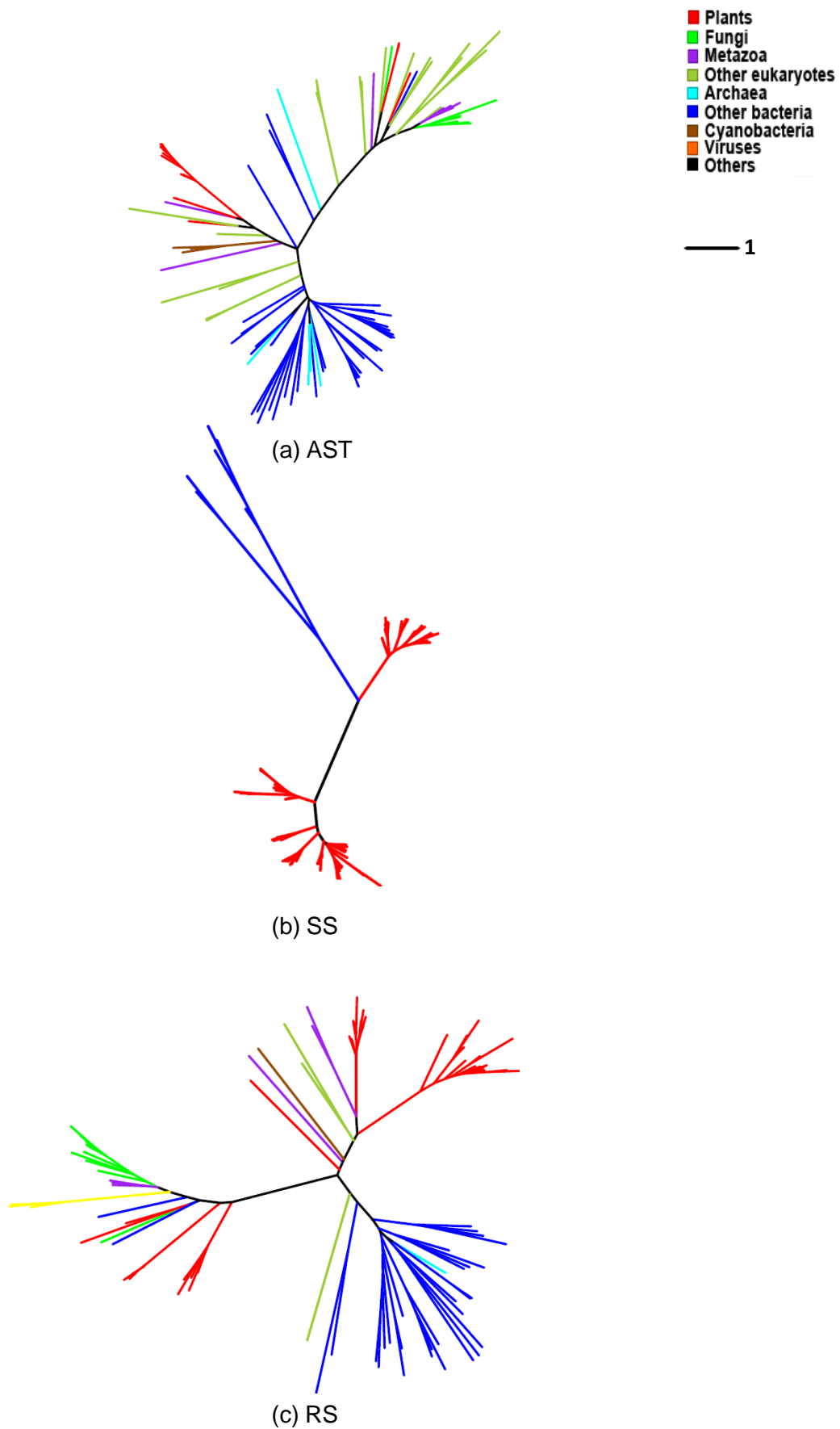

Figure S2: Phylogenetic trees of 100 GT8 sequences sampled by (a) AST, (b) SS and (c) RS, respectively.

Supplement: Figure S3 — Phylogenetic trees of 100 GT8 sequences sampled by AST, SS, and RS respectively. See the legend of Figure 3 for further details. (PDF) [file pone.0098844.s003.pdf]

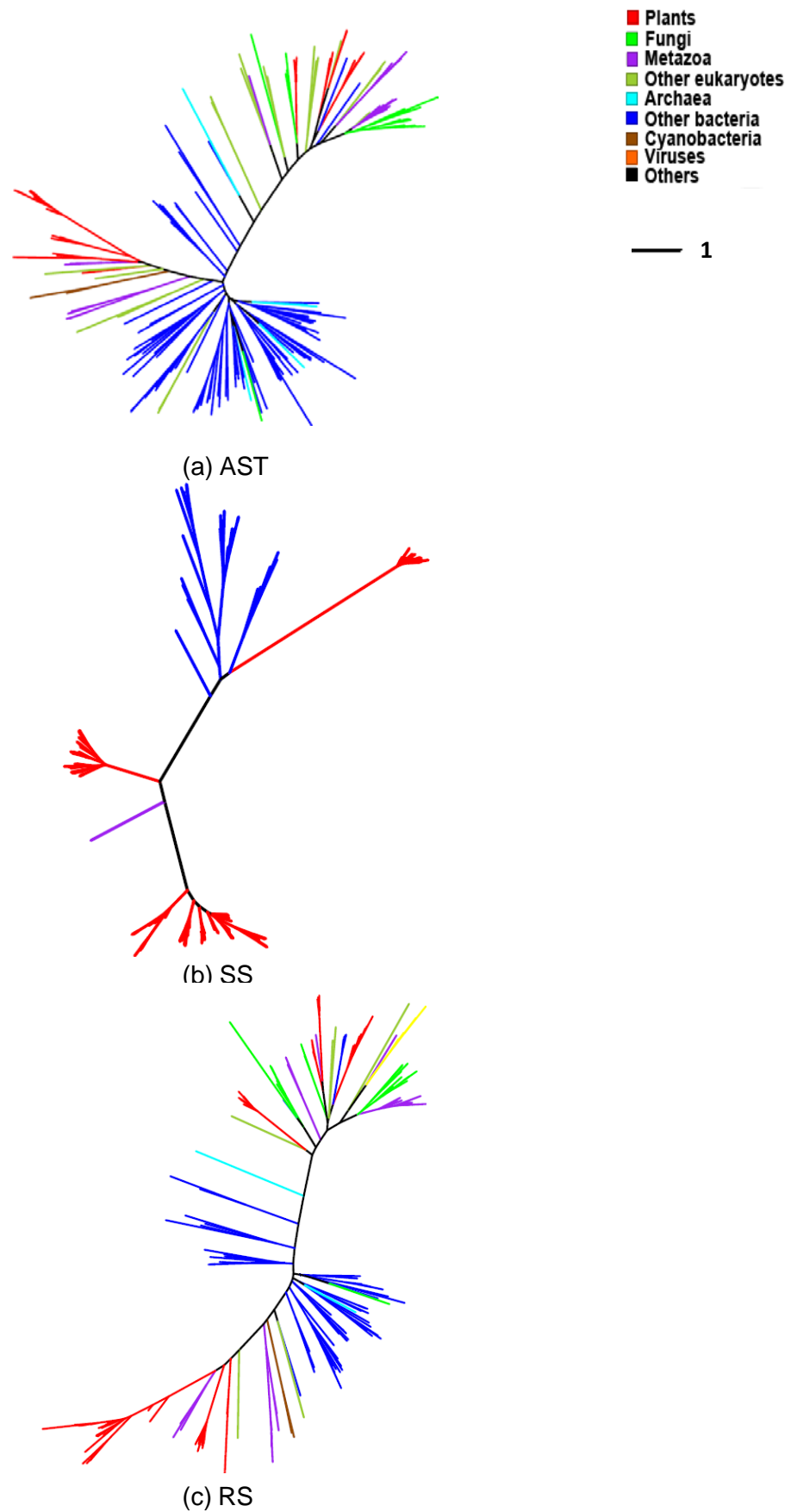

Figure S3: Phylogenetic trees of 200 GT8 sequences sampled by (a) AST, (b) SS and (c) RS, respectively.

Supplement: Figure S4 — Phylogenetic trees of 200 GT8 sequences sampled by AST, SS and RS respectively. See the legend of Figure 3 for further details. (PDF) [file pone.0098844.s004.pdf]

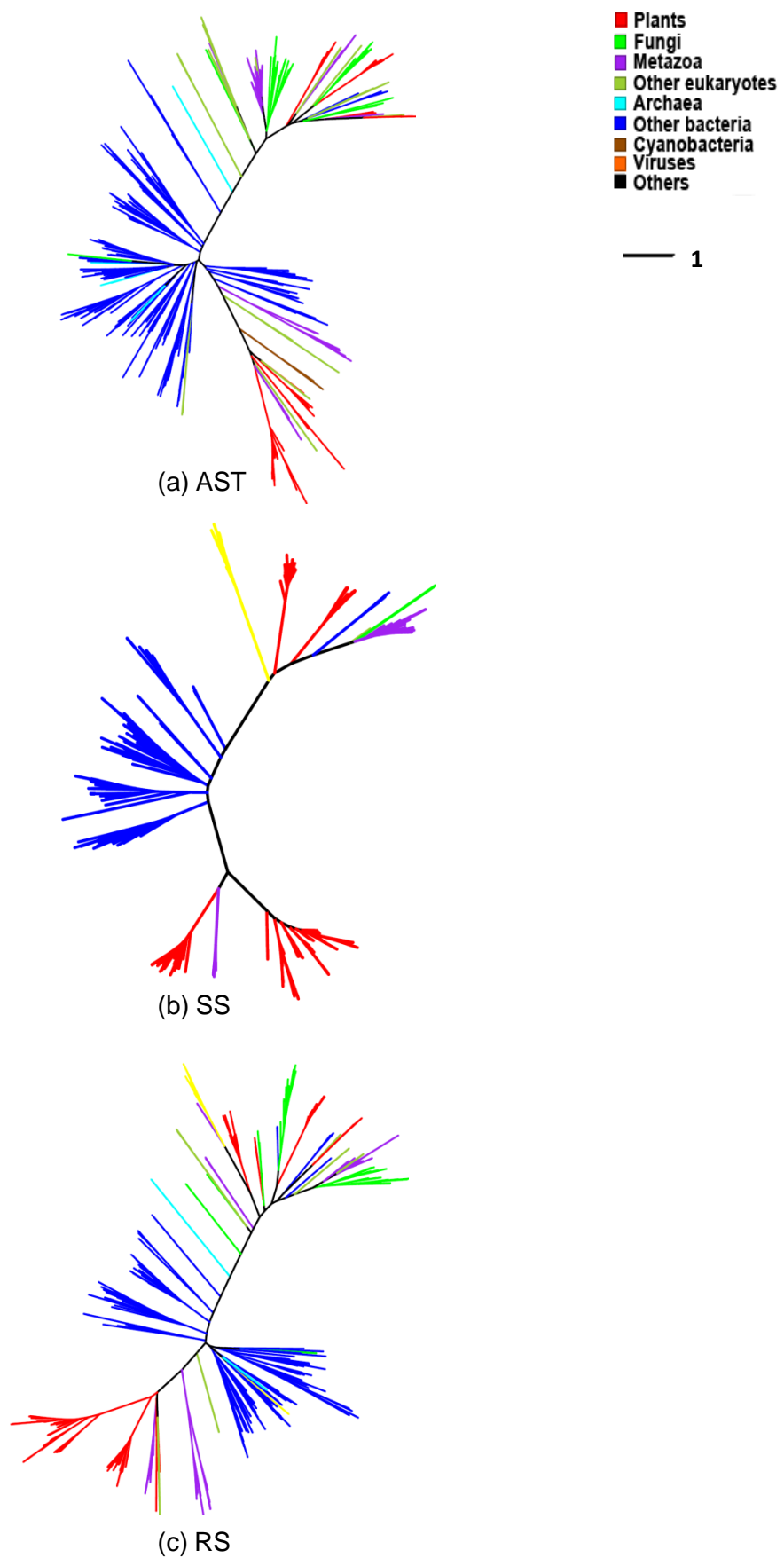

Figure S4: Phylogenetic trees of 400 GT8 sequences sampled by (a) AST, (b) SS and (c) RS, respectively.

Supplement: Figure S5 — Phylogenetic trees of 400 GT8 sequences sampled by AST, SS and RS respectively. See the legend of Figure 3 for further details. (PDF) [file pone.0098844.s005.pdf]
